# Psychiatric Disorders Differently Correlate with Physical Self-Rated Health across Ethnic Groups

**DOI:** 10.3390/jpm7040006

**Published:** 2017-11-13

**Authors:** Shervin Assari

**Affiliations:** 1 Department of Psychiatry, School of Medicine, University of Michigan, Ann Arbor, MI 48109, USA; assari@umich.edu; Tel.: +1-734-647-7944; Fax: +1-734-763-7379; 2 Center for Research on Ethnicity, Culture and Health, School of Public Health, University of Michigan, Ann Arbor, MI 48109, USA

**Keywords:** ethnic groups, psychiatric disorders, self-rated health

## Abstract

In this study, we compared 10 ethnic groups for associations between psychiatric disorders and physical self-rated health (SRH) in the United States. Data came from the Collaborative Psychiatric Epidemiology Surveys (CPES), 2001–2003. The study included 7587 non-Latino White, 4746 African American, 1442 Mexican, 1106 other Hispanic, 656 other Asian, 600 Chinese, 577 Cuban, 520 Vietnamese, 508 Filipino, and 495 Puerto Rican individuals. The Composite International Diagnostic Interview (CIDI) was used to measure psychiatric disorders, including major depressive disorder (MDD), general anxiety disorder (GAD), social phobia, panic disorder, post-traumatic stress disorder (PTSD), alcohol abuse, and binge eating disorders. A single-item measure was used to estimate physical SRH. Demographic (age and gender) and socioeconomic (education and income) factors were also measured. Unadjusted and adjusted correlations between psychiatric disorders and physical SRH were calculated. Major ethnic variations were found in the correlation between psychiatric disorders and physical SRH; as well as the role of demographic and socioeconomic status (SES) factors in explaining these associations. non-Hispanic Whites, Cubans, and African Americans showed more correlations between psychiatric disorders and physical SRH than other ethnic groups. In non-Hispanic Whites, the associations between psychiatric disorders and physical SRH were explained by demographic factors. In African Americans, the link between psychiatric disorders and poor physical SRH were explained by SES indicators. In conclusion, although single-item physical SRH measures are traditionally assumed to reflect the physical health needs of populations, they may also indicate psychiatric disorders in some ethnic groups, such as non-Hispanic Whites, Cubans, and African Americans. Demographic and socioeconomic factors also have differential roles in explaining the link between psychiatric disorders and physical SRH. Physical SRH does not exclusively reflect physical health, and it may be more biased by mental health across some ethnic groups.

## 1. Introduction

Single-item self-rated health (SRH), which measures the overall health of individuals [1], is a useful screening tool for estimating the health needs of populations [2,3,4]. Single-item SRH predicts a wide range of physical, mental, and behavioral health outcomes [2,5,6,7,8,9,10,11]. Whether or not single item SRH measures similarly reflect health needs across diverse ethnic groups is still unknown [12,13,14,15,16,17,18,19,20,21,22,23,24,25,26].

As SRH may not have the same meaning across ethnic groups [17], it is plausible to expect cross-ethnic variation in the association between psychiatric disorders and physical SRH [17]. Although we know that mental SRH better reflects mental problems, and physical SRH better reflects physical health problems [12,13,14,15,16,17,26], very few studies have ever tested the mental health correlates of physical SRH, particularly across various ethnic groups. While the literature has shown that overall SRH reflects psychological distress [27] and psychiatric disorders [5,6,9,10,11], very limited knowledge exists on how racial and ethnic groups differ in the mental health correlates of physical SRH. The current study compares 10 ethnic groups for the associations between psychiatric disorders and physical SRH in the United States.

The meaning of poor SRH may be under the influence of ethnicity and culture [9,16,17,25]. As a result, the health correlates of SRH may vary across diverse populations [12,16,27]. Recent literature suggests that single-item SRH measures may be more sensitive to the health needs of non-Hispanic Whites than any other ethnic groups [9]. However, very limited information is known about the comparability of single-item SRH, as they reflect mental [28] and physical health [13,14,28,29,30] across ethnic groups.

Ethnic groups may differ in the process of somatization, which is defined as the expression of emotional distress as physical rather than psychological symptoms [31]. Some research suggests that ethnic minorities have a higher tendency to somaticize their mental health issues [32,33]. Minority populations that have low access, trust and high stigma toward mental health care systems may express and perceive their mental and emotional symptoms as due to physical health problem [18]. This somatization may be one of the mechanisms that explain the lower mental health care utilization of ethnic minorities [34]. Due to higher somatization, we can expect the association between psychiatric disorders and physical SRH to be stronger in ethnic minority groups.

This study explored cross-ethnic variation in the associations between psychiatric disorders and physical SRH in the United States.

## 2. Methods

### 2.1. Design and Setting

This was a secondary analysis of the Collaborative Psychiatric Epidemiology Surveys (CPES), 2001 to 2003. The CPES is composed of the National Latino and Asian American Study (NLAAS), the National Survey of American Life (NSAL), and the National Comorbidity Survey Replication (NCS-R). These three surveys are representative of United States adults and have employed very similar methodologies, including utilizing trained lay interviewers to conduct interviews primarily in person. Data were collected by the Institute for Social Research at the University of Michigan in Ann Arbor. Study design and sampling have been described in detail elsewhere [35].

### 2.2. Participants

This current study included a national household probability sample of 18,237 individuals including 520 Vietnamese, 508 Filipino, 600 Chinese, 656 other Asian, 577 Cuban, 495 Puerto Rican, 1442 Mexican, 1106 other Hispanic, 4746 African American, and 7587 non-Latino Whites. All participants were adults (aged 18 or older). These numbers came from NLAAS (*n* = 4649), NSAL (*n* = 6082), and NCS-R (*n* = 9282).

### 2.3. Ethics

The study protocol was approved by the University of Michigan Institutional Review Board. Participants received financial compensation for participating in this study.

Informed consent was obtained from all individual participants included in the study. All procedures performed in studies involving human participants were in accordance with the ethical standards of the institutional and/or national research committee as well as the 1964 Helsinki declaration and its later amendments, or comparable ethical standards.

### 2.4. Interview

Most interviews were face-to-face and conducted within participants’ homes. A minority of the interviews were conducted via phone. The average response rate of the CPES is 72.7%.

### 2.5. Measures

#### 2.5.1. Race and Ethnicity

Race and ethnicity in the CPES was measured by the individual’s self-identification. Participants self-identified as Asian, Hispanic, Black/African American, or White/Caucasian. Asians then self-identified as Vietnamese, Filipino, Chinese, or other Asian. Hispanics identified as Cuban, Puerto Rican, Mexican, or other Hispanic. Blacks identified as African American or Caribbean Blacks [36].

#### 2.5.2. Physical Self-Rated Health

Participants were asked “How would you rate your overall physical health?” Responses included five categories: excellent, very good, good, fair, and poor. Single-item physical SRH measures correlated with several aspects of health and well-being. Test–retest reliability for single-items is high, ranging from 0.7 to 0.8 for brief time intervals [30]. These single-item measures also correlate with detailed standard multi-item scales [30]. A review showed that in 23 of 27 studies, SRH was associated with mortality above and beyond the effect of age and socioeconomic status, and in several studies, chronic conditions and medical risk factors as well [2].

#### 2.5.3. Demographic Factors

Demographic factors including age (continuous measure) and gender (dichotomous measure, males being the reference category) were measured.

#### 2.5.4. Socioeconomic Characteristics

Socioeconomic factors including education level—less than high school (reference category), high school graduate, some college, college graduate—and income (continuous measure) were measured.

#### 2.5.5. Lifetime Psychiatric Disorders

A modified version of the World Mental Health Composite International Diagnostic Interview (WMH-CIDI) was used to evaluate lifetime major depressive disorder (MDD), general anxiety disorder (GAD), social phobia, alcohol abuse, binge eating disorders, panic disorder, and post-traumatic stress disorder (PTSD). All disorders were diagnosed based on the Diagnostic and Statistical Manual of Mental Disorders, Fourth Edition (DSM-IV). The WMH-CIDI was originally developed for the World Mental Health project initiated in 2000 [37]. The CIDI requires trained lay interviewers to generate diagnoses of lifetime and recent DSM-IV-/ICD-10 disorders [38]. Clinical reappraisal studies have documented good concordance for CIDI diagnoses with diagnoses made by psychiatrists [37,38,39,40]. The CIDI has shown to be valid among several ethnic groups [41,42,43].

### 2.6. Statistical Analysis

To account for the complex sampling design of the CPES, we used Stata 13.0 (Stata Corp., College Station, TX, USA) for data analysis. Standard errors were estimated using the Taylor series approximation technique. We conducted our analyses within each ethnic group. For bivariate associations, zero-order Pearson correlations were calculated. For multivariable associations, we calculated partial correlations.

First, we estimated zero-order correlations by calculating the Pearson’s correlation coefficient. Then, we used partial correlation tests while age and gender were controlled. Finally, we used partial correlation tests while age, gender, education, and income were controlled. In all analyses, physical SRH was treated as a continuous measure, with a higher score indicating worse health. Psychiatric conditions were all dichotomous variables. Due to multiple comparisons, we used a more conservative threshold for our *p*-values, where *p* of less than 0.01 was considered statistically significant.

## 3. Results

### 3.1. Descriptive Statistics across Ethnic Groups

Table 1 provides a summary of demographic and socioeconomic status (SES) characteristics across 10 ethnic groups in the study. Table 1 also describes the sample sizes of each ethnic group. As shown in the table, the largest population was composed of non-Latino Whites (41.6%). Our population was representative of over 200 million individuals in the United States. Age was lowest in Mexicans and highest in Cubans followed by non-Latino Whites. Education was lowest in Mexicans, followed by African Americans. Income was lowest among African Americans, followed by Mexicans (Table 1).

### 3.2. Unadjusted Associations

Table 2 provides a summary of bivariate correlates of physical SRH across ethnic groups. Major ethnic differences were found in correlates of physical SRH. Particularly in three ethnic groups, namely, non-Hispanic Whites, Cubans, and African Americans, several psychiatric disorders were positively correlated with physical SRH, suggesting that the presence of psychiatric disorders is associated with worse physical SRH (Table 2).

### 3.3. Partially Adjusted Associations

Table 3 summarizes adjusted correlations between psychiatric disorders and physical SRH when age and gender are controlled for. This table also shows major ethnic differences in correlations between psychiatric disorders and physical SRH. Age and gender explained why several psychiatric disorders were correlated with physical SRH in non-Hispanic Whites, but not in Cubans or African Americans. That is, in non-Hispanic Whites, psychiatric disorders and physical SRH were not correlated net of age and gender. However, Cubans and African Americans showed associations between psychiatric disorders and physical SRH above and beyond age and gender (Table 3).

### 3.4. Fully Adjusted Associations

Table 4 shows the results of adjusted correlations when demographic factors and SES are controlled. As shown in this table, SES explained the correlation between psychiatric disorders and physical SRH in African Americans, but not in Cubans. In African Americans, psychiatric disorders were not correlated with physical SRH net of demographic and SES indicators. In Cubans, psychiatric disorders were still correlated with physical SRH above and beyond demographic and SES indicators (Table 4).

## 4. Discussion

This study was conducted to compare various ethnic groups for the associations between psychiatric disorders and physical SRH in the United States. In line with our hypothesis, major ethnic-group differences were found. We also found cross-ethnic variations in the role of demographic and SES factors in explaining the association between psychiatric disorders and physical SRH.

In African Americans, Cubans, and non-Hispanic Whites, a wide range of psychiatric disorders were associated with physical SRH. In African Americans, SES explained the association between psychiatric disorders and physical SRH. In non-Hispanic Whites, however, age and gender explained the associations between psychiatric disorders and physical SRH. In Cubans, neither demographic nor SES explained the link between psychiatric disorders and physical SRH.

Our findings can be explained by the literature on ethnic differences in the degree to which mental disorders are somaticized [32]. Non-White ethnic groups such as Hispanics [32], African Americans [44], and East Asians [33] may have a higher tendency for somatization of mental health symptoms than non-Hispanic Whites. Studies have shown that women in general [32], and ethnic minority women in particular [45,46,47], have a high tendency for somatization of psychiatric disorders such as depression. Future research should explore how the intersection of race, ethnicity, class, and place shape somatization.

In countries such as United States, ethnic minorities may use somatization to cope with their mental health issues, which is defined as the diversion of emotional distress to physical rather than psychological symptoms. In the presence of somatization, patients experience and report bodily complaints due to psychiatric disorders [32]. In a study comparing clinical samples of Thai and American children, Thai children were reported to have higher levels of somatic versus depressive symptoms relative to American children [33]. Ethnicity, gender, and SES all influence the degree to which an individual is emotionally expressive [32]. We argue that differences in somatization, emotional expressivity, and stigma explain cross-ethnic variation in associations between psychiatric disorders and physical SRH.

Our findings extend the literature differential validity of SRH across ethnic groups [33,44,48]. Recent research has documented considerable ethnic differences in the association between SRH and psychiatric disorders [9,16,17]. SRH differently reflects past, current, and future health needs across ethnically diverse individuals [27,49], and not only ethnic differences: SES differences also exist in the reliability and validity of SRH [50]. Thus, one reason SRH reflects the various health domains of ethnic groups is their SES differences. However, in this study, we did not control for SES differences between ethnic groups, only the SES variation within each ethnic group. We need to conduct more studies on the cumulative and interactive effects of ethnicity and SES on meanings of SRH. As SRH has differential validity for measuring “true” health statuses across ethnic groups [22,24], and SRH reflects different aspects of health across ethnic groups [16,17,18], SRH should not be simply used for the comparison of health status across groups. So, SRH may not be an ideal tool to measure health disparities across ethnic groups. There is a need for more validation studies of SRH and mental health measures [12,13]. There is also a need to study the cognitive and emotional mechanisms behind ethnic differences in regards to how actual health needs are reflected in the perception and report of SRH [9,12,14,23,27,30,51,52,53,54].

We also found ethnic group variation in the confounding role of demographic and SES factors on the associations between psychiatric disorders and physical SRH. In non-Hispanic Whites, age and gender explained the associations between psychiatric disorders and physical SRH. This means that age and gender had a more salient effect in shaping both mental (e.g., psychiatric disorders) and physical (e.g., SRH) health in non-Hispanic Whites. However, for African Americans, it was not age and gender, but rather low SES that explained why psychiatric disorders and physical SRH were linked. In Cubans, psychiatric disorders and physical SRH did not correlate because they were more common in demographic or SES groups. The role of demographic factors in the health of Whites [55,56,57], and role of SES in the health of Blacks [58,59,60] are well-known. In fact, low SES at least in part explains why Blacks experience worse health outcomes in United States [59,60,61]. Research has shown that demographic and SES factors differently determine physical and mental SRH across ethnic groups [25]. Previous research has also shown that demographic and SES factors differently confound the associations between physical and mental health outcomes across various populations [20,58,62,63,64,65].

Our findings have implications for clinical and public health practices with ethnically diverse populations. Solely relying on single-item SRH measures will result in differential levels of bias in the measurement of health across various ethnic groups [15,66]. Single-item SRH should accompany more comprehensive measures if we wish to compare health and illness across ethnically diverse populations [24]. Currently, SRH is being used as a screening tool to enroll populations for interventions [5,24]. SRH is also being employed to measure the efficacy of an intervention both in clinical and community settings. As said above, we recommend the use of other measures in addition to SRH to provide more nuanced information on the actual health status of ethnically diverse populations [66].

Although we showed ethnic differences in how psychiatric conditions influence the evaluation of one’s physical health, the mechanisms behind these group differences are still unknown. In addition, it is not clear how biases in the measurement of health status of diverse populations can be reduced [9]. There are more questions than answers when it comes to the interplay of ethnicity, gender, class, and health perception [17].

This study had a few limitations. CIDI may be differentially valid for the diagnosis of psychiatric disorders across ethnic groups. The study did not measure whether participants had received a psychiatric diagnosis by a clinician, and whether they had chronic medical conditions. The current study did not use a comprehensive list of psychiatric disorders. As single-item SRH measures are sensitive to the contextual effects of preceding questions in surveys [30], other researchers may find different results if they use other surveys. Despite all these limitations, the current study used a nationally representative data with a very large sample size.

To conclude, various ethnic groups differ in their associations between psychiatric disorders and physical SRH. Cross-ethnic variations also exist in the role of demographic and SES factors in explaining the association between psychiatric disorders and physical SRH.

## Figures and Tables

**Table 1:** Descriptive statistics across ten ethnic groups.

	Vietnamese		Filipino		Chinese		Other Asian		Cuban		Puerto Rican		Mexican		Other Hispanic		African American		Non-Latino Whites	
Characteristics	M	SE	M	SE	M	SE	M	SE	M	SE	M	SE	M	SE	M	SE	M	SE	M	SE
Age (Years)	43.73	0.67	42.98	0.75	42.88	0.61	38.10	0.68	48.97	0.73	41.17	0.72	36.68	0.48	38.38	0.52	42.19	0.27	46.73	0.45
Education (Rank)	2.33	0.05	2.92	0.05	2.90	0.05	3.24	0.04	2.39	0.05	2.14	0.05	1.82	0.03	2.25	0.04	2.28	0.02	2.69	0.02
Income (USD1000)	51.25	2.18	79.01	2.54	74.32	2.56	76.07	2.59	52.22	2.25	50.52	2.18	41.40	1.30	49.43	1.54	37.12	0.54	61.72	1.08
*N*	520	508	600	656	577	495	1442	1106	4746	7587	520	508	600	656	577	495	1442	1106	4746	7587
Weighted *n*	1,156,292	1,909,580	2,533,495	3,452,027	1,060,586	1,864,484	15,763,471	5,869,754	22,049,686	1.48 × 10^8^	1,156,292	1,909,580	2,533,495	3,452,027	1,060,586	1,864,484	15,763,471	5,869,754	22,049,686	1.48 × 10^8^
%	2.85	2.79	3.29	3.6	3.16	2.71	7.91	6.06	26.02	41.6	2.85	2.79	3.29	3.6	3.16	2.71	7.91	6.06	26.02	41.6

M: Mean; SE: Standard error.

**Table 2:** Unadjusted correlations between physical self-rated health (SRH) and psychiatric disorders across 10 ethnic groups.

	Vietnamese	Filipino	Chinese	Other Asian	Cuban	Puerto Rican	Mexican	Other Hispanic	African American	Non-Hispanic Whites
MDD	0.194 *	0.066	0.030	0.115	0.232 *	0.174 *	0.129 *	0.154 *	0.152 *	0.178 *
GAD	0.174 *	0.090	−0.053	0.077	0.229 *	0.100	0.001	0.106 *	0.034	0.156 *
Social Phobia	0.123 *	0.082	0.068	0.081	0.178 *	0.175 *	0.075	0.116 *	0.113 *	0.128 *
Panic	0.135 *	0.056	−0.051	0.083	0.189 *	0.142 *	0.040	0.076	0.118 *	0.097 *
PTSD	0.058	0.001	0.015	0.128 *	0.183 *	0.093	0.052	0.084	0.101 *	0.144 *
Alcohol	0.013	0.115 *	−0.078	0.070	0.009	0.014	0.074	−0.020	0.092 *	0.116 *
Eating Disorders	0.092	0.036	−0.042	0.123 *	0.077	0.099	0.040	0.077	0.088 *	0.123 *

MDD: Major depressive disorder; GAD: General anxiety disorder; PTSD: Post-traumatic stress disorder; * *p* < 0.01.

**Table 3:** Partially adjusted correlations between physical self-rated health SRH and psychiatric disorders across ten ethnic groups.

	Vietnamese	Filipino	Chinese	Other Asian	Cuban	Puerto Rican	Mexican	Other Hispanic	African American	Non-Hispanic Whites
MDD	0.208 *	0.086	0.044	0.126 *	0.239 *	0.176 *	0.132 *	0.149 *	0.208 *	0.086
GAD	0.143 *	0.089	−0.059	0.071	0.216 *	0.105	−0.017	0.086	0.143 *	0.089
Social Phobia	0.137 *	0.090	0.100	0.082	0.190 *	0.192 *	0.072	0.112 *	0.137 *	0.090
Panic	0.122 *	0.060	−0.057	0.085	0.176 *	0.144 *	0.039	0.104 *	0.122 *	0.060
PTSD	0.040	−0.003	0.011	0.126 *	0.195 *	0.097	0.047	0.028	0.040	−0.003
Alcohol Disorder	0.029	0.131 *	−0.060	0.091	0.061	0.038	0.079	0.026	0.029	0.131 *
Eating Disorders	0.098	0.043	−0.038	0.131 *	0.075	0.123 *	0.045	0.028	0.098	0.043

MDD: Major depressive disorder; GAD: General anxiety disorder; PTSD: Post-traumatic stress disorder; * *p* < 0.01. Age and gender are controlled.

**Table 4:** Fully adjusted correlations between physical self-rated health (SRH) and psychiatric disorders across ten ethnic groups.

	Vietnamese	Filipino	Chinese	Other Asian	Cuban	Puerto Rican	Mexican	Other Hispanic	African American	Non-Hispanic Whites
MDD	0.206 *	0.079	0.044	0.128 *	0.217 *	0.134 *	0.139 *	0.145 *	0.212 *	0.126 *
GAD	0.137 *	0.095	−0.054	0.073	0.194 *	0.086	−0.019	0.083	−0.004	0.140 *
Social Phobia	0.137 *	0.105	0.098	0.087	0.183 *	0.164 *	0.077	0.124 *	0.145	0.113 *
Panic Disorder	0.120 *	0.058	−0.065	0.086	0.177 *	0.115	0.032	0.094	0.052	0.071
PTSD	0.026	0.000	0.022	0.128 *	0.179 *	0.069	0.053	0.038	0.030	0.140 *
Alcohol Use Disorder	0.029	0.130	−0.065	0.092	0.057	0.047	0.082	0.036	0.020	0.093 *
Binge Eating	0.094	0.051	−0.021	0.131 *	0.074	0.101	0.048	0.029	−0.024	0.045

MDD: Major depressive disorder; GAD: General anxiety disorder; PTSD: Post-traumatic stress disorder; * *p* < 0.01. Age, gender, education, and income are controlled.
